# Wombs, Worms and Wolves: Constructing Cancer in Early Modern England

**DOI:** 10.1093/shm/hku039

**Published:** 2014-06-16

**Authors:** Alanna Skuse

**Keywords:** cancer, malignancy, caustics, surgery, Galen, zoomorphism, canker

## Abstract

This essay examines medical and popular attitudes to cancer in the early modern period, *c*.1580–1720. Cancer, it is argued, was understood as a cruel and usually incurable disease, diagnosable by a well-defined set of symptoms understood to correspond to its etymological root, *karkinos* (the crab). It was primarily understood as produced by an imbalance of the humours, with women being particularly vulnerable. However, such explanations proved inadequate to make sense of the condition's malignancy, and medical writers frequently constructed cancer as quasi-sentient, zoomorphising the disease as an eating worm or wolf. In turn, these constructions materially influenced medical practice, in which practitioners swung between anxiety over ‘aggravating’ the disease and an adversarial approach which fostered the use of radical and dangerous ‘cures’ including caustics and surgery.

In 1714, Daniel Turner, a London physician, published his only work exclusively on diseases of the skin: *De Morbis Cutanels*. Turner discussed rashes, carbuncles, warts and imposthumes of many varieties, but most striking was his account of an extraordinary story from a ‘villainous Empiric’ who claimed to have cured a woman with a cancerous ulcer:

Such an [tall tale] I was not long since inform'd of, by a Woman who vow'd, that in Time of Dressing, one of these Ulcers, by a villainous Empiric (a famous Cancer Doctor) when they held a Piece of raw Flesh at a Distance from the Sore, the Wolf peeps out, discovering his Head, and gaping to receive it.^1^

What motivated a ‘famous’ doctor to make such a claim, and a gentleman-physician such as Turner to repeat it? Why should a wolf be discovered in a cancerous ulcer, and how was ‘raw Flesh’ supposed to remedy the situation? This essay examines the neglected topic of cancer in the early modern period, and argues that this is a malady which demonstrates, perhaps more clearly than any other, the contemporary reciprocity between imaginative constructions of disease and the pragmatic experience thereof. Cancer was a disease which terrified patients and medical practitioners alike, and in the charged discourses surrounding it, one can see how experiences of the disease, from diagnosis to treatment, were both mediated by existing cultural beliefs about the humours, gender and illness, and contributed to the development of a pervasive early modern image—the devouring, duplicitous ‘canker’.

Turner's 1714 text comes almost at the end of the period I wish to examine, 1580–1720. His work, like most of those explored in this essay, was an instructional medical text, printed for the benefit of those studying and practising medicine as well as those with a layman's interest in the subject. However, this essay also looks to domestic receipt books, casebooks, letters, plays and poems in an attempt to gain as rounded a picture as possible of how potential sufferers, as well as those involved in treating the disease, apprehended their condition. Turner's text was printed in the vernacular, making it accessible to a wide audience of fellow practitioners and lay people. Elsewhere, the essay makes use of medical texts which were translated into English from French, German, Dutch or Latin. Though this essay examines those texts' impact on practice in England, it is clear that there was a high degree of consanguinity in ideas about cancer between Britain and mainland Europe.^2^ The long seventeenth century is chosen for several reasons. It invites inspection of how attitudes to the disease changed, or failed to change, during a period in which medical historians have noted change and conflict in medical theory, regulation and professionalization.^3^ Moreover, it is a time frame just long enough to yield sufficient surviving material for an informed look at what appears to have been an uncommonly diagnosed disease.

This period is also one in which the history of cancer has remained largely unexplored, in marked contrast to the nineteenth and twentieth centuries. In the past three decades, several historians of medicine have attempted to pinpoint the first incidence of cancer, and have made the case for the disease's presence in ancient Egyptian, Indian or Mesopotamian texts, without exploring if or how cancer may have been identified in those cultures.^4^ More recently, the most comprehensive twenty-first-century works on the history of cancer, Siddhartha Mukherjee's *The Emperor of all Maladies*, and James S. Olson's *Bathsheba's Breast*, devote barely a handful of pages between them to the conceptualisation and treatment of the disease between Hippocrates, in the fourth century bc, and John Hunter, nearly 2000 years later in the mid-eighteenth century.^5^ Luke Demaitre's insightful article on the topic has filled in many of the gaps for the medieval period, focusing in particular on cancer's supposed relationship to leprosy.^6^ In early modern studies, however, investigations into the history or ‘meanings’ of cancer have remained relatively limited, in both number and scope. Building on literary-focused analyses of ‘canker’ by Lynette Hunter and Jonathan Gil Harris, Sujata Iyengar's *Shakespeare's Medical Language* recognizes ‘canker’ as a term which denoted a bodily complaint as well as horticultural blight, and briefly describes typical symptoms of cancer.^7^ However, in common with Hunter and Harris, Iyengar downplays the differentiation between cancer and other forms of skin disease, and the correlation between dramatic figurations of ‘canker’ and zoomorphic understandings of cancerous disease. Several recent works by medical historians have also drawn attention to the plight of early modern cancer sufferers. In her 2012 *Female Patients in Early Modern Britain*, for instance, Wendy Churchill recognizes that the early modern history of breast cancer has been ‘subsumed’ into broader chronologies of the disease.^8^ She briefly describes the common symptoms of and treatments for breast cancer, and contends that this was a disease of which most early modern women were aware.^9^ A similar, and equally concise, description can be found in Stolberg's *Experiencing Illness*, in which he identifies cancer as ‘ranked among the diseases which aroused the greatest fear’ in the early modern period, on account of the pain and ‘massive physical decline’ it effected.^10^

Undoubtedly the most comprehensive work on early modern cancer to date, however, is Marjo Kaartinen's recently published *Breast Cancer in the Eighteenth Century*.^11^ Kaartinen's text discusses the supposed causes of, and methods of diagnosis for, cancer, but focuses in particular on breast cancer therapies, both pharmaceutical and surgical, and on the physical experiences of women undergoing these treatments. Although her work informs this essay, Kaartinen's approach to cancer emphasises scientific innovation, particularly in the later eighteenth century, while paying relatively little attention to those who, in the earlier part of the century, continued to position the disease within a humoral framework. Moreover, Kaartinen's text, like those of Churchill and Iyengar, focuses on the physical rather than cultural experience of this disease: the symptoms of cancer, and its curative and palliative treatments. In contrast, as I shall describe, this essay dwells upon conceptualisations of the disease as a zoomorphic, quasi-ontological entity during the ‘long’ seventeenth century, and reflects on how those conceptualisations influenced medical practice.

Understanding more fully how early modern people thought about cancer is significant for several reasons. The study of cancer has obvious implications for scholars of early modern literature, in which (with the exception of Iyengar's work) the ubiquitous ‘canker’ has, as I discuss below, repeatedly been glossed as referring to a botanical parasite, without taking into consideration its medical resonance. More broadly, cancer provides cultural historians and historians of medicine with an opportunity to trace the elision of figurative and literal in early modern experiences of illness, reading medical texts, and medical practice, as both shaping and shaped by the anthropomorphic and zoomorphic images with which people attempted to make sense of this frightening, painful and usually fatal disease. This essay begins by considering whom cancer was supposed to affect, and what was believed to be its cause. In the second part, I consider the ‘nature’ of cancer: the symptoms by which it was diagnosed, the relationship between cancer and canker, and the conceptualisation of the disease as parasitical, even sentient. The final section considers how medical practitioners explained the malignancy of cancer, and how characterisations of the disease as ‘rebellious’ and ‘evil’ affected attempts at cure.^12^

## Framing Cancer's Victims: Discussions of Pathology and Cause

What *was* cancer, for a person encountering that term in the early modern period? A 1707 text by the French chirurgeon Pierre Dionis, published in an English translation in 1710, suggested that ‘A Cancer is universally agreed to be the most terrible of all the evils which attack Mankind’.^13^ ‘Though Wars and Plagues kill in less time,’ he admitted, ‘they don't yet, to me, seem so cruel as the Cancer, which as certainly, though more slowly, carries those afflicted to the Grave, withal causing such Pains as make them every day wish for Death.’^14^ Cancer was, then, a much feared disease, worthy of the apprehension of an eminent practitioner such as Dionis. Further, he pronounced,

'Tis a Disease which attacks not only the Breast, but several other Parts, on which it is not less outrageous: It sometimes assumes different names; when it comes on the Legs, 'tis called the Wolf, because if left to itself, 'twill not quit them 'till it has devoured them; when it fixes on the Face, 'tis called a Noli me tangere, because that touching irritates it, and makes it a greater Ravage: Authors also observe, that there are besides Tumours and cancerous Ulcers in several parts of the Body, which I shall not mention to Day.^15^

Dionis' text tells us something of the difficulties as well as the rewards of examining cancer. This disease, he explains, is principally found in the breasts, although it also invades ‘several parts’. From the earliest ‘formal’ identifications of cancer in ancient Greek texts, this practical restriction, of being unable to see into living bodies and detect internal tumours, had been similarly acknowledged.^16^ Moreover, cancer ‘attacks’ the sufferer, can be irritated, and ‘devours’ flesh; that is, it is a disease with personality, understood as somehow separate from the cancer patient. Finally, it ‘assumes different names’, each of which may emphasise a different facet of the disease's pathology, and which make the complaint difficult to trace through historical sources. These indeterminacies present considerable difficulties for the medical historian. However, an era's interest in, or insistence upon, certain names for and characteristics of the disease may also signal points of particular interest around which one can view contemporary constructions of cancer as being arranged. Mukherjee asserts that ‘Rolling underneath … medical, cultural, and metaphorical interceptions of cancer over the centuries was the biological understanding of the illness’.^17^ For early modern people, I argue, the connection was still more intimate. Beliefs about the disease's cause show that the medical *was* also the cultural and metaphorical.

To diagnose cancer, one first had to know where to look, and for early modern medical practitioners, this seems to have been a straightforward decision. As Dionis admitted, though the disease could strike ‘several parts’ it was, paradigmatically, a malady of the female breasts. The anonymous 1670 *An Account of the Causes of Some Particular Rebellious Distempers* declared that ‘Cancers are known in part by the Places they fix on … a Cancer may happen to sundry Places, as the Lips, Tongue, Cheeks, Womb, and other loose Glandulous Parts; but were [sic] One has a Cancer in any part besides, Twenty have them in their Breasts.’^18^ Likewise, James Handley's 1705 *Colloquia Chirurgica* advised that ‘Altho' it is possible for it to breed in all Parts of the Body, yet [cancer] generally seizes either the *Breasts* or *Matrix* of Women, and the Lips and Nose of the Face.’^19^ Reasons for the striking bias toward diagnosis of breast cancers over any other were manifold. Breasts presented a visible, palpable, accessible site: while Lazarius Riverius admitted that ‘al kinds of Tumors may arise in the Stomach as wel as other parts’, internal tumours were seldom mentioned, being largely un-diagnosable and, it was agreed, incurable.^20^ The sole exception to this rule was the womb, where cancers were occasionally diagnosed either by digital or instrumental examination, or more commonly, by the fetid discharge or ‘sanies’ expelled from that organ.^21^ Furthermore, in the eyes of many practitioners, the breasts were composed of such a material, and part of such a body—namely, the cold, moist female body—that their vulnerability to cancers was exponentially greater than any other part. The breasts, it was argued, were ‘spungy’, ‘laxe’ and ‘funguous’.^22^ Their tissues, seemingly less dense than those elsewhere on the body, were correspondingly more inclined to soak up bad humours. As the authors of *The Compleat Midwife's Practice* attested, ‘The Canker proceeds from a feculent and grosse humour … which when nature cannot void, it most commonly in women empties itself upon the breast, by reason of this cavernous and spongy nature.’^23^ Medical practitioners' admission that cancer could occasionally strike elsewhere—for example, on the face—rather confirmed than undermined their theory, since other at-risk spots, such as nose, lips and ears, had in common the same fleshy ‘looseness’.^24^

The female body also provided a humoral pathology which seemed designed to foster cancers. As Gail Kern Paster and others have shown, women's bodies paradigmatically occupied the ‘cold and wet’ quarter of the humoral spectrum.^25^ Lacking ‘vital heat’, women did not concoct a portion of their blood into seed, as men did, or concocted only watery, thin seed, and instead this excess nutrition went either to the nourishment of a fetus, or was expelled through the menses.^26^ When menstruation was interrupted for any reason other than pregnancy, therefore, women found themselves particularly vulnerable to a build-up of excess, excremental humours, especially melancholy. Such humours commonly gathered in the womb, from whence they were supposed to be expelled, and physicians explained incidences of womb cancer with reference to this fact.^27^ Moreover, the womb was believed by many to have a direct connection to the breasts, such that, as Marylynn Salmon has observed, the post-partum absence of menstruation was taken as proof that excess blood was being transformed into milk for the nursing child.^28^ John Sadler, for example, insisted in 1636 that breast milk was ‘nothing but the menstruous bloud made white in the breasts’, having been altered in order to avoid the alarming sight of infants covered in blood, while in 1657, a translated text by the French physician and surgeon Jean Riolan (following the venerated opinion of Leonardo Da Vinci), posited the existence of two veins by which the breasts and womb were directly connected.^29^ That connection meant that humours from the womb were bound to migrate to the breasts over any other part of the body.

Medical practitioners were broadly united in their belief that a surfeit of humours in the affected area was the precursor of cancer. In particular, writers on the subject usually held that melancholy, or black bile, was chiefly to blame. Robert Bayfield's pronouncement in the 1662 *Tractatus de Tumoribus* was typical: ‘when this melancholious humor, resembling in proportion the dregs of wine, doth descend and flow into any member, and there abideth compact together … sometimes it breedeth a Cancer, as when the same is somewhat cool'd.’^30^ As Bayfield's description of the ‘dregs’ of melancholy indicated, the substance's implication in cancers was symptomatic of its broader characterisation as feculent, malign, and even associated with the devil.^31^ In his study of Renaissance self-identity, *Sources of the Self*, Charles Taylor argues that ‘black bile doesn't just cause melancholy [i.e. melancholia]; melancholy somehow resides in it. The substance embodies this significance.’^32^ Just as the properties of melancholy humour were realised in the disease of melancholia, the malignancy and evil associated with this substance were also, as we shall see, manifest in descriptions of intractable, cruel and rebellious cancerous tumours.

Despite the humour's negative associations, however, few practitioners argued that melancholy in itself caused cancers. After all, a healthy bodily complexion included all four humours, each with positive as well as dangerous attributes. Instead, many practitioners shared Bayfield's view that melancholy became dangerous when it gathered in one place, where it ‘compacted’ or became ‘stagnant’. Again, this belief traded on the popular image of melancholy humours as excremental dregs. Thomas Nashe's 1594 *The Terrors of the Night*, for example, characterised ‘the thick steaming fenny vapours’ of melancholy as like ‘slime and dirt in a standing puddle’, likely to engender ‘misshapen objects’.^33^ Still more practitioners viewed cancer as the result of a qualitative change in the melancholy humour provoked either by heat or by combination with other substances, particularly choler. Alexander Read, who (unsuccessfully) treated Nicholas Culpeper's mother for cancer in 1639, proposed in 1635 that cancerous tumours might arise ‘from *Atra bilis*, or melancholy, or choler adust … for there are two sorts of *Atra bilis:* the one is caused of naturall melancholy adust: the other is caused of yellow choler burned, and it is much more maligne than the former.’^34^ This view was one inherited from medieval accounts of cancerous disease, and it remained prevalent well into the eighteenth century.^35^ Almost 70 years after Read's pronouncement, John Browne asserted in his 1703 *The Surgeons Assistant* that ‘a *Scirrhus* is made by natural Melancholy, which is in the Blood … but a Cancer is not bred from natural, but adust [burned] Melancholy’.^36^ Adust melancholy was, as Paster has observed, a substance deemed even more toxic than regular black bile, and it was described as unnatural, foreign, malignant and degenerate by contemporary physicians.^37^ In common with much early modern medicine, however, explanations of cancer frequently concluded at this point, offering only vague or partial descriptions of how, exactly, malignant humours caused malignant tumours. To explain cancer's frightening propensity to overtake the body, humoral theory was set aside, and medical practitioners of all kinds employed explanatory models which blurred the line between metaphor and somatic reality.

## ‘Knowing’ Cancer: Symptoms and Characterisations of Malignant Disease

Early modern patients were subject, as Olivia Weisser has shown, to any number of lumps and bumps.^38^ Cancer, however, was recognisable by a very distinct set of symptoms, primarily visual in form and intimately tied to perceptions of the disease's unique ‘nature’. Medical practitioners generally, though not universally, agreed that cancerous tumours were accompanied by heat and pain. As Christof Wirsung asserted, ‘the Canker causeth … great paine and beating, whereof *Schirrhus* is free’.^39^ Others, including Paré, described ‘a sense like the pricking of Needles’.^40^ Also characteristic of the disease was a distinctive and discomfiting appearance. Appearing under the skin ‘From the smalness of a Vetch [legume] to the bigness of a Pomion [fruit—often an apple]’, tumours were often highly coloured, either red and ‘livid’ or ‘blackish’.^41^ Unlike some other swellings, cancers were said to be ‘rough and unequall’ and ‘round’; that is, circular, but with an uneven surface appearance.^42^ The most distinctive mark of a cancerous tumour, however, was the appearance of darkened veins extending from the swollen area. A ‘Canker in the Breasts’, argued *The Compleat Midwife's Practise*, ‘is known by the crooked windings, and retorted veins that are about it, stretching out long roots a good way from it, being sometimes blackish, and sometimes inclined to black and blue’, while Dionis noted that the disease was ‘Remarkable … on account of the Veins of black Blood disperst over its whole Superfices’.^43^ Such symptoms announced the presence of a cancer far before the disease's most feared stage, the ‘breaking out’ of a morbid cancerous ulcer, ‘inequall, sordide, turned over, cavernous, evill favoured’ and ‘round horrible’.^44^

The visual characteristics of cancerous tumours proved easy ones for medical writers and their audiences to remember. Not only were they peculiarly gruesome even by the standards of the age, but, crucially, they evoked the very name of the disease, a derivation of the Greek *karkinos*, or crab. Round and red, the tumour appeared like the body of that creature, whilst the blood vessels extending outward were ‘verie like unto the feete of crabbes, descending from the round compasse of their bodies’.^45^ For early modern as for medieval authors, the comparison seemed a perfect one, ‘exquisite’ in its fit to the disease's symptoms.^46^ According to Read's *Chirurgicall Lectures*:

as a crab, in Latine *Cancer*, hath a body and feet of a livid colour, and whatsoever it claspeth with the clawes, it holdeth it firmly, so this griefe is of a livid colour, and so girdeth the part which it possesseth, that it seemeth to be nailed to the part, and about it the full veines exquisitely imitate the feet of a crab: and from these similitudes the tumor hath its name.^47^

From the etymology of cancer, one can see how the term became used interchangeably with ‘canker’ across many texts, medical and otherwise, across the early modern period. The ejective form, ‘canker’, hearkened back to the Latin *cancer* (both the ‘c's being pronounced ejectively), which was a stepping stone between Greek and English terms.^48^ ‘Cancer’ and ‘canker’ were both used, seemingly indiscriminately, to describe the disease we now recognise as cancer. Interestingly, however, ‘canker’ could also be used to describe a broader range of ulcers, including mouth ulcers and pox sores, which, judging from their description and treatment in contemporary texts, were clearly not confused by medical practitioners with the more serious malignant ulcers and tumours of ‘true’ cancer. Such a confusion of linguistic forms naturally presents a challenge to the medical historian, and even sometimes frustrated medical practitioners of the period, with the Swiss physician Théophile Bonet complaining of the ‘Cheat and Errour’ made by his contemporaries with regard to these terms.^49^ However, as Read's observation demonstrates, the evolution of ‘cancer’ as a term also shows how closely the disease was associated with its namesake, both in behaviour and form. Not only did they look alike, but, as Peter Lowe asserted, cancer ‘gnaweth, eateth and goeth like this fish [the crab]’.^50^ Moreover, cancer was, according to Philip Barrough, ‘verie hardly pulled awaie from those members, which it doth lay holde on, as the sea crabbe doth, who obstinately doth cleave to that place which it once hath apprehended’.^51^

The attribution of zoomorphic behaviours to cancerous tumours was central to their designation as ‘crab-like’. It can also help to explain the disease's monikers of ‘worm’ and ‘wolf’, and in particular, why the ‘canker-worm’, commonly read by scholars as denoting an eating caterpillar, had such an intimate relationship to another kind of parasite—the ‘worm’ of cancer. Cancers, as Lowe and many others observed, appeared to ‘gnaw’ and ‘eat’ the sufferer.^52^ Tumours became larger as the patient visibly diminished. Moreover, like a parasite, cancers were often only discovered by their appearance at the body's surface after the patient had been suffering pain and emaciation from the disease for some time. These distinctive factors marked cancer out from the multitude of degenerative diseases to which one might fall victim in the period, and gave rise to a zoomorphic idea of the disease unmatched elsewhere in the medical lexicon. This phenomenon bears striking similarities to the construction of pain as recently identified by Joanna Bourke. In ‘What is Pain?’, Bourke argues that metaphorical statements, such as ‘a pain *in* my shoulder’, are apt to be ‘literalised’, such that we fall for an ‘ontological fallacy’ of believing pain to have an independent existence. In descriptions of cancerous disease, this ‘literalising’ is taken still further.^53^ Harris has observed, in his analysis of Gerard Malynes' 1601 *A Treatise of the Canker of England's Common Wealth*, that the worm image lent ‘ontological agency’ to the ‘eating’ action of cancer.^54^ Employing this observation in service of an analysis of economic language, Harris does not pursue the worm analogy through its progress from figural tool to medical belief. However, the primary materials show that at various points throughout the sixteenth, seventeenth and eighteenth centuries, some medical practitioners became convinced not only of the *likeness* of cancer to parasites, but of the disease's literal composition as such. In one such example from William Salmon's 1687 *Paraieremata*, it was reported that

A certain Emperick did cure many Cancers by this one medicine: He took Worms, called in Latin centum pedes, in English Sowes; they are such as lye under old Timber, or between the Bark and the Tree. These he stamped and strained with the Ale, and gave the patient to drink thereof morning and evening. This medicine caused a certain Black Bug or Worm to come forth, which had many legs, and was quick, and after that the Cancer did heal very quickly with convenient Medicines.^55^

Elsewhere, this conviction was repeated in various forms. Both physicians and receipt book writers offered remedies to ‘slea the worme’, with Elizabeth Sleigh and Felicia Whitfield advising that ‘For a canker in a Womans breast’, one should ‘Take goose, sallandine [celandine] bray them together well … lay them to the dugge or teat it will cleanse the canker, kill the worme, and heale the sore.’^56^ Many more practitioners included crushed or powdered worms and insects in their cancer remedies, in hopes of curing like with like.^57^ Presenting an account similar to that of the ‘certain Empirick’, but with a veneer of scientific respectability, Dionis declared in 1710 that

Some believe, that the ulcerated Cancer is nothing else but a prodigious Multitude of small Worms, which by little and little devour all the flesh of the part: What made room for this Opinion, is, that with the Microscope we have sometimes discerned some of these Insects in Cancers; and that putting a bit of Veal on the Ulcer, the Patient has felt less Pain; because, say they, these Worms then feeding on the Veal, leave the Patient at rest for some time.^58^

The cure Dionis describes was a popular palliative cure for cancer throughout the early modern period, and, like many cancer therapies, had its roots in medieval practice.^59^ Applying fresh meat, whether veal, poultry, or, less commonly, puppies and kittens, was felt to offer the eating cancer something more tempting to consume, affording temporary respite to the patient.

The particular emphasis on cancer's appetite for meat also gestured to another of the disease's pseudonyms, the wolf. As Turner's account of the creature emerging from the body demonstrates, this image seeped into popular use as a byword for an eating tumour by dint of its ‘ravenous’ and ‘fierce’ characteristics.^60^ Naturally, it was harder to imagine this creature as literally rather than metaphorically present in the body: nonetheless, some practitioners (including Turner's ‘villainous Empiric’) evidently did just that, representing the wolf as ‘discovering his Head’ from within a cancerous ulcer. Elsewhere, the inclusion of powdered ‘wolves-tunge’ in a cancer remedy from Oswald Gabelkover's 1599 *The Boock of Physicke* likewise testifies to the real therapeutic impact which even apparently figurative zoomorphism wrought upon treatment for cancer.^61^ ‘Wolf’ could be used to describe a cancer anywhere on the body, but was, as Dionis observed, most commonly used to designate tumours and ulcers on the legs. Why this should have been the case is unclear, and may have gestured toward wolves' *modus operandi*, seizing the hind legs of their prey. In any case, it appears that this comparison had been in circulation for many years. In 1363, for example, the French surgeon-physician Guy de Chauliac linked cancer's ‘wolfishness’ with ‘meat cures’ such as that later described by Dionis, grimly suggesting that ‘the people say that [cancer] is called “wolf”, because it eats a chicken every day, and if it did not get it it would eat the person’.^62^

Zoomorphic metaphors for cancer traded on the disease's symptoms, and materially affected how cancerous tumours and ulcers were treated. In aligning cancer with worms and wolves, medical writers also accessed long-standing cultural, religious and scientific discourses about those creatures. Wolves, for example, were described in the Bible as ‘ravening’ animals, and, moreover, creatures liable to appear in sheep's clothing.^63^ As Karen Edwards notes, contemporary writers of poetry and polemic frequently utilised those tropes to vilify certain groups, such that ‘The figurative wolf in Milton's works consistently represents those with Romish allegiances or inclinations, promoters of superstition, arch-hypocrites, and rapacious predators’.^64^ The analogy was lent particular force by the fact that wolves, like Catholics, had been eliminated from England by the sixteenth century, but still roamed in continental Europe, and were felt as a constant threat. Similarly, Bruce Thomas Boehrer observes that wolves became a popular symbol of deception in early modern culture, augmented by the presence of three wolf fables in William Caxton's influential 1483 edition of Aesop.^65^ The *Oxford English Dictionary* locates the first use of ‘wolf’ to describe fierceness or rapacity even earlier, in the *c*.950 Lindisfarne Gospels (and only around 175 years after the word's first recorded use *c*.725).^66^ It seems likely that such abiding discourses of expulsion and corruption both bolstered, and later drew from, the eating ‘wolf’ of cancer, and by the early seventeenth century, preachers and dramatists were using ‘wolf’ in ways which clearly showed their understanding of the word's medical context. Henry ‘Silver-tongue’ Smith, for example, informed his congregation in the late sixteenth century that ‘[covetousness is] … like the disease which we call the Wolfe, that is always eating, and yet keeps the bodie leane’.^67^ In John Webster's 1612 *The White Devil*, the villainous Flamineo refers to ‘meat cures’, as outlined above, when he describes himself as ‘like a wolf in a woman's breast … fed with poultry’ (5.3.54).^68^

The ‘worm’ of cancer, meanwhile, drew even more extensively on diverse contemporary and ancient discourses about bodily parasites. The presence of intestinal worms in children, and maggots in unstitched wounds, had long fostered the notion that worms of several varieties could live in the body's cavities. Moreover, as Matthew Cobb describes, it was commonly believed in the early modern period that worms could be spontaneously generated by decaying organic matter.^69^ With the rise of microscopy in the mid-seventeenth century, interest in bodily worms reached new heights, with several texts providing lurid accounts of worms found in various parts of the body.^70^ Indeed, Turner, who had related (and discounted) the tale of the wolf emerging from a cancerous ulcer, asserted in 1714 that

That not only Worms of sundry kinds, but other living Creatures are found in our Bodies (however they come there) is too notorious to want Proof: Nay, that our Blood is full of them, that most of our Diseases take Rise from them, more especially the Cancer, Itch, Ringworm, *&c.* has been asserted by learned Men.^71^

Literary and biblical sources, meanwhile, continually positioned worms as a *memento mori* associated in various ways with man's sinfulness and bodily frailty. Linguist and classicist Calvert Watkins has noted the prevalence of ‘slaying the worm’, particularly in relation to illness, as a ‘mythographic basic formula’ in a number of ancient Proto-Indo-European languages.^72^ Closer to home, worms also appear in the Bible on numerous occasions as a reminder of the mortality of the flesh, with Job declaring that ‘though after my skin worms destroy this body, yet in my flesh shall I see God’ (19:26).^73^ The presence of worms in the body after death was therefore an example of the transience and corruptibility of the flesh, a theme on occasion expanded to include viewing oneself as a lowly worm.^74^ Intriguingly, worms also appeared in literature from the medieval period onward as agents of conscience, physically ‘gnawing’ at the minds, hearts, and bowels of sinners in a manner uncannily akin to breast-devouring cancer worms.^75^ ‘Like a worm to gnaw the heart,’ as one treatise observed, conscience provokes ‘our own thoughts to trouble and affray.’^76^ As creatures both generated by the self and hostile to it, ‘eating’ one in a figurative and a painfully literal sense, gnawing conscience-worms seem to have been influenced by images of gnawing bodily worms, and such images no doubt contributed in turn to the popularisation of a parasitical vision of cancerous disease.

## Cancer as *Dramatis Persona* in Prognosis and Treatment

Possible conflict between viewing cancer as a parasitical disease or a disease of the humours seems to have gone unremarked in the early modern period. Descriptions of the cancer worm often appeared alongside explanations of *atra bilis*, while in remedies for the disease, crabs, worms and wolves' tongues shared the page with simples for correcting choler and melancholy. That this was possible attests to the flexibility which characterised much early modern thought on disease causation. It also indicates the conceptual utility of a permeable boundary between figural and literal notions of cancer. Notably, there was no suggestion from either ‘villainous Empiric[s]’ or licensed physicians that the worm or wolf of cancer entered the body from without.^77^ Nonetheless, conceptualising cancerous tumours as somehow distinct from the patient in whom they were situated allowed both practitioners and patients to make sense of the disease's seemingly unstoppable progress and resistance to cure.

Medical practitioners of the early modern period keenly observed the growth of cancerous tumours, their transformation into ulcers, and on occasion, their metastasis to other parts of the body.^78^ A 1651 edition of Nicholas Culpeper's popular *Directory for Midwives*, for example, delineated the progress of breast cancer as ‘a little tubercle, no bigger than a pease, [which] … grows up by degrees, and spreads out roots with Veins about it’.^79^ Other practitioners dwelt upon the disease's possible degeneration into ‘an ulcer round horrible’, or, less commonly, its capacity to ‘break forth afresh, either in the same place, or in some other parts of the Body’.^80^ Sometimes, medical writers tried to explain these phenomena as evidence of the poisonous nature of cancers, or their relation to contagious diseases, primarily leprosy and venereal pox.^81^ For the most part, however, it was agreed that this mysterious phenomenon was simply an inextricable part of cancer's nature. ‘Malignancy’ in its fullest sense was the defining characteristic of cancer, connecting the otherwise puzzling ‘behaviours’ of the malady to a semi-sentience that was universally understood as evil, cruel and deliberately intractable. As one anonymous medical practitioner put it, ‘[it] discovers its evil Nature by the grievous Symptoms that appear, and a[s] it increases in bigness, it increases in malignity.’^82^

Casting cancer as a *dramatis persona* apart from the sufferer allowed for the evolution of ‘cancer’ and ‘canker’ as particularly useful terms for describing maladies of rhetorical bodies in which one part seemed to rebel against the rest, such as duelling among the aristocracy, corruption in parliament, or ‘Civil dissension’, the ‘viperous worm / That gnaws the bowels of the commonwealth’.^83^ It also contributed to a discourse which viewed medicine as having the moral and professional task of defeating this ‘malignant and stubborn enemy’, however difficult and dangerous that task might be.^84^ The most common treatments for cancer during this period appear to have been relatively gentle ones. The majority of medical practitioners began their prescriptions by urging patients to stick to a spare, ‘cooling’ diet and avoid strong, salty foods or potent wines.^85^ In addition, more or less mild purgatives, analgesics such as nightshade and henbane, and plants thought to quell flux, expel melancholy, and strengthen the body all appear frequently in the tisanes, broths, unguents and salves prescribed in most medical texts.^86^ This circumspect approach reflected the contemporary view that most cancers were incurable, and worse, were ‘enraged’ by the application of harsh medicines.^87^ In line with images of the disease as ravenous, punitive and semi-sentient, many believed that, as Barrough stated, ‘the malignitie of the evill through … vehement medicines is stirred, and provoked, and made more fierce and savage’.^88^ Hippocrates' aphorism 6.38 was often cited in support of instituting only palliative cures: ‘it is better not to cure occult, or hidden *Cancers*; for the Patients cured … doe quickly die, but such as are not cured live longer’.^89^

Despite such cautions, however, the therapies most strongly associated with cancer were not gentle, holistic, palliative cures. Frequently decried in medical textbooks, but nonetheless described with grim fascination, chemical caustics and surgery were the most radical courses available to the cancer patient and their practitioner. In these cures, the removal of the cancer from the body by any means necessary took centre stage, despite the massive risks this posed to the patient. Arsenic and mercury, which had been in use as mortifying agents since the medieval period, accomplished this feat as ‘caustics’ or ‘septics’. Applied to the cancer site, they would, in theory, break the skin over the tumour, and mortify the flesh of the resulting ulcer so that it died and sloughed off or could be cut away. In practice, they frequently killed the patient. In 1684, Bonet recorded that:

I have observed [septics], especially Arsenick … applied to Ulcers near the heart, as to a Cancer in the breast, that they once carried off a Woman in 6 days: About three hours after the Powder was strewed on her Breast, she … was taken with a Shivering, then with a Vomiting, and frequent Faintings, with a languid Pulse; which symptomes, encreasing by degrees, her extreme parts growing cold, and her Face and whole Body swelling beyond measure, she was miserably murthered.^90^

As Bonet's account makes clear, such therapies were hazardous to the patient, but they also posed economic and moral risks to the medical practitioner, who risked being cast as a ‘murderer’.

This risk was exponentially increased in relation to surgery for cancers, most notoriously the mastectomy operation. In this procedure, the breast was excised from the body, without anaesthesia, using a sharp wire or a knife, and the area usually cauterised with a hot iron.^91^ It was clearly a horrific experience, with an extremely high mortality rate. According to Browne, ‘you at once had as well cut your Patients Throat, as use the Knife in this case, by which you will certainly see her fall under your hands, and in her goar Blood make her exit’.^92^ Nonetheless, mastectomies, along with less invasive operations to remove surface tumours, continued throughout the early modern period.^93^ For patients experiencing debility, social isolation, and constant pain, facing the prospect of a slow death from their illness, the seemingly extraordinary decision to undergo surgery was sometimes deceptively straightforward. In his 1686 *Several Chirurgical Treatises*, for example, Wiseman recorded one patient's response to being informed of the risks of surgery: ‘I had rather die than live thus.’^94^

Both surgeons and patients were uncomfortably aware of the mortal danger which accompanied cancer surgeries. Unsurprisingly, therefore, not all surgeons were willing to undertake such hazardous procedures, which posed a substantial risk to their reputation and therefore their livelihood.^95^ Of those who did, some implied that the experience was traumatic for the operator as well as the patient. In 1687, for example, a text by Read and another unnamed author, *Chirurgorum Comes*, warned that for all tumour operations, surgeons needed to be particularly ‘resolute, chearful in countenance and speech, and no ways scrupulous’.^96^ After observing the mastectomy operation of ‘Mrs Townsend’ in the mid-seventeenth century, the Reverend John Ward recorded that ‘One of the chyrurgeons told her afterwards, that shee had endured soe much, that hee would have lost his life ere hee would have sufferd the like.’^97^ Despite these scruples, however, the undertaking of these operations, and the language in which they were recorded, implies that conceptualisations of cancer as evil and alien were central to the desire among physicians, patients, and especially surgeons, to aggressively remove tumours and ulcers. The 1585 *A Compendious Chyrurgerie*, for example, counselled surgeons to ‘fetch it out, roote and all, with instrumentes or causticke medicines: to wit, cutte it wholly awaye’.^98^ Over a century later, John Moyle similarly pronounced: ‘This Cancer, or wolf, cannot be cured any other Way than by extirpating of it.’^99^

The adversarial language in which some surgeons represented cancer operations thus added to early modern people's widespread ambivalence toward medical practitioners in general and surgeons in particular.^100^ Cancer operations, in which the body was dangerously and visibly altered, put surgeons at risk of being deemed avaricious and reckless, with the operator characterised, as in one 1703 text, as ‘[a] shameful Undertaker, who makes no more of taking off a Breast (altho’ no otherwise than a Butcher might do the same) than some Persons do to pair [pare] their Nails'.^101^ At the same time, however, cancer surgeries represented the limits of medical intervention, and the fact that it was surgery, not physic, which seemed to offer the best chance of a cure for cancer may have contributed to the growing sense among some surgeons that their trade was a noble and professional one, on a par with that of the physician.^102^ The most innovative surgeons of the period flocked to view and partake in radical cancer operations, seeking new ways in which to minimise patients' blood loss and prevent the enemy from returning.

## Conclusion

As a (diagnosed) cause of death in the early modern period, cancer was a minor player. As a disease which played upon the consciousness of Englishmen and women throughout the sixteenth, seventeenth and eighteenth centuries, however, it had a prominent part on the cultural stage, and remains of major significance to social, medical and cultural historians. Examination of the supposed causes, symptoms and cures for cancer show how the way in which this disease was experienced was of a piece with how it was imagined. Moreover, two things remained remarkably stable over a period which saw sea-changes elsewhere in medical understanding. Constructions of cancer as both of and distinct from oneself, generated by the body and hostile to it, materially affected the development of treatments for the malady, while the pragmatic facts of the disease's symptoms and response to ‘cures’ reciprocally contributed to its reputation as cruel and rebellious. Like those who came before and after them, early modern people feared cancer, and were only slightly less afraid of the treatments which could supposedly relieve it. On 3 December 1700, Lady Sarah Cowper wrote in her diary: ‘My Breast is unquiet and gives me troublesome apprehensions. I sometimes seem weary of living, yet find myself often in fear of a painfull lingering Death.’^103^ The marginal note alongside the entry reads ‘Fearing a Cancer’, and Cowper's apprehension was heightened by the death of several female acquaintances from the disease.^104^ It was against this backdrop of anxiety and indeterminacy that the seemingly incredible tale of the wolf ‘discovering his head’ from a cancerous ulcer found a readership in the early eighteenth century. Whether by appeal to troublesome female bodies, feculent humours, or ravening worms and wolves, early modern medical writers and their audiences united scholarship, observation, and imagination as they sought a way to understand how their bodies could so dramatically turn against them.

## Funding

‘This work was supported by the Wellcome Trust [SL-04755].
